# Internet-delivered cognitive–behaviour therapy for anxiety related to asthma: study protocol for a randomised controlled trial

**DOI:** 10.1136/bmjresp-2023-002035

**Published:** 2024-05-27

**Authors:** Marianne Bonnert, Stephen Nash, Erik M Andersson, Sten Erik Bergström, Christer Janson, Catarina Almqvist

**Affiliations:** 1 Centre for Psychiatry Research, Dep of Clinical Neuroscience, Karolinska Institutet, Stockholm, Sweden; 2 Region Stockholm, Stockholm, Sweden; 3 Department of Medical Epidmiology and Biostatistics, Karolinska Institutet, Stockholm, Sweden; 4 Department of Clinical Neuroscience, Karolinska Institutet, Stockholm, Sweden; 5 Pediatric Allergy and Pulmonology Unit at Astrid Lindgren Children’s Hospital, Karolinska University Hospital, Stockholm, Sweden; 6 Department of Medical Sciences: Respiratory Medicine, Uppsala University, Uppsala, Sweden

**Keywords:** Asthma, Complementary Medicine, Psychology

## Abstract

**Introduction:**

There is an established association between asthma and anxiety. The overlap between asthma symptoms and symptoms of anxiety may cause individuals to overestimate their asthma severity and restrict their daily activities leading to a low quality of life. There is currently weak evidence for treatments targeting anxiety related to asthma, but cognitive–behavioural therapy (CBT) has shown some promising but mixed results. The current randomised controlled trial will investigate if exposure-based internet-delivered CBT (Internet-CBT) is more effective than treatment as usual+medical education (TAU+ME) to relieve symptoms of anxiety and asthma control.

**Methods and analysis:**

90 participants will be randomised 1:1 to 8 weeks of Internet-CBT or TAU+ME. The primary outcome, the patient-reported Catastrophising Asthma Scale, will be analysed from baseline to the primary endpoint at 16 weeks using hierarchical linear mixed model of the slope over time. Secondary outcomes, such as asthma control, quality of life and forced expiratory volume in 1 s, will be analysed correspondingly.

**Ethics and dissemination:**

All participants will be informed about the study and leave their consent before study entry. All results will be analysed at group level and reported through publication in a peer-reviewed scientific journal within the field. The study received ethical approval by the Swedish Ethical Review Authority in January 2020 (ID: 2019-05985; 2022-01117-02).

**Trial registration number:**

Registered at ClinicalTrials.gov (ID: NCT04230369).

**WHAT IS ALREADY KNOWN ON THIS TOPIC**

- Symptoms of anxiety may complicate asthma perception. Cognitive–behavioural therapy (CBT) targeting the asthma-anxiety connection is promising, but the evidence base is weak.

**WHAT THIS STUDY ADDS**

- Internet-CBT may be efficacious to relieve symptoms of anxiety and perceived asthma control.

**HOW THIS STUDY MIGHT AFFECT RESEARCH, PRACTICE OR POLICY**

- Internet-CBT can be distributed regardless of geographical distances. If efficacious, a novel evidence-based treatment may be widely made available to individuals suffering from anxiety related to asthma.

## Introduction

Individuals with asthma frequently experience troublesome symptoms such as wheezing, dyspnoea, hoarseness and tightness in the chest. Interestingly, some of these symptoms are also associated with anxiety.^1^ Research has indicated that anxiety disorders are twice as common in people with asthma compared with the general population,^2–5^ and asthma patients with comorbid anxiety tend to have poorer asthma control, reduced quality of life, lower medical adherence and increased healthcare utilisation.^6–8^ Treatment guidelines have acknowledged the need to detect and target anxiety in asthma but there is currently weak evidence for psychosocial treatments,^9^ although cognitive–behavioural therapy (CBT) has shown some promising results.^10^ A Cochrane review that included nine trials saw some support for CBT as an intervention for asthma.^11^ However, there was a great variability in study quality and large drop-out rates. Thus, the authors conclude that the evidence is currently weak, and even the most optimal form of CBT delivery needs further investigation.^11^

### Models for understanding aetiology of anxiety-related asthma

Studies on CBT for anxiety and asthma often consist of multicomponent packages including psychoeducation about asthma and optimal medication use, differentiation between asthma and panic symptoms, progressive relaxation, cognitive restructuring, problem solving, exposure to feared stimuli, smoking reduction interventions and assertiveness training.^12–14^ A potential issue with multicomponent CBT packages is the absence of a clear hypothesis regarding potential treatment mechanisms. As a result, the components included in the intervention may lack theoretical coherence, which can decrease efficacy and offer little direction for future improvement.^15^ Although some components may be effective, the training provided for each component may not be sufficient to support cognitive or behavioural changes in participants. Thus, it may be worthwhile to explore the possibility of enhancing CBT by developing a more condensed treatment model that specifically incorporates a coherent framework for the learning and maintenance of anxiety-related asthma.

Experimental studies have demonstrated that asthma patients often have an inaccurate perception of respiratory symptoms and that anxiety seems to have a negative influence on the ability to distinguish between symptoms of asthma and other bodily sensations.^16 17^ This can make it challenging for asthma patients with heightened anxiety to properly distinguish valid threats (eg, tobacco smoke, allergens, cold air and respiratory tract infections) from safe situations (eg, travel by public transport, physical activity and talk in public).^18^ As a consequence, many may avoid situations unnecessarily, leading to excessive restrictions in daily functioning and a diminished quality of life.^19^ Moreover, the process of generalisation can cause symptoms similar to asthma to be perceived as a threat, further exacerbating fear and avoidance behaviours.^20^

Thus, there may be a behavioural pattern of fear and avoidance in anxiety-related asthma similar to the pattern observed in anxiety disorders, where fear of situations and stimuli associated with anxiety may lead to excessive avoidance.^21^ The behavioural avoidance perpetuates the fear, and a vicious cycle is formed. The gold standard to treat anxiety disorders is exposure-based CBT.^22^ Exposure-based CBT has demonstrated efficacy for several somatic disorders where fear of symptoms may lead to excessive avoidance.^23–28^

### Internet-delivered CBT facilitates broad dissemination

Even if CBT is effective, there is a well-known treatment gap between the need for treatment and the availability of evidence-based treatments.^29^ A recent development within the CBT field has been to deliver the treatment over the internet. Manualised treatments are easy to transfer to online patient material. Participants can login to the internet-delivered CBT (Internet-CBT) at their own convenience, read the material online and plan their weekly exercises in online worksheets. Guided Internet-CBT use therapists trained in Internet-CBT that read and comment through short written messages on the participant’s progress in the online platform. Because the material presented online contains everything that a CBT therapist often explains verbally in traditional CBT, therapist time is saved. Internet-CBT thus could potentially increase the availability of CBT and can be delivered over any geographical distance.^30^ As such, Internet-CBT has the potential to provide specialised care for disorders that few therapists are trained to treat.^31^ Studies of Internet-CBT have been performed for several somatic and psychiatric disorders, with treatment effects similar to those obtained in studies of face-to-face CBT.^32^

### Previous results

Our research group has previously developed and evaluated exposure-based CBT for anxiety related to asthma. In a previous study, a treatment protocol was developed and tested on six participants evaluated with multiple baselines and weekly measurements throughout treatment.^33^ The participants reported a very high level of satisfaction with the intervention, and we found evidence of improvement in all important clinical outcomes (eg, asthma-related anxiety and worry). The standardised treatment protocol was adapted to Internet-CBT and evaluated in a feasibility study where all participants (N=31) received Internet-CBT.^34^ Mean adherence to Internet-CBT was good (80% of all content), and most participants (89%) reported adequate relief after treatment. Catastrophising Asthma Scale (CAS) improved substantially (Cohen’s d=1.52, p<0.001) from pretreatment to the primary endpoint 2 months after treatment completion. All except one secondary outcome, including asthma control, avoidance behaviours, Fear of Asthma Symptoms Scale (FAS) and quality of life, improved with moderate to large effect sizes (Cohen’s d=0.67–1.44, all p<0.01). In the final secondary outcome (quality of life, BBQ), we observed a small non-significant improvement at 2 months (Cohen’s d=0.40, 95% CI −0.11 to 0.92) but strong evidence of an improvement over time (slope 0.49 points on the BBQ scale per week; 95% CI 0.18 to 0.80). All improvements were stable at 4 months follow-up. In conclusion, Internet-CBT is feasible and has the potential to provide substantial relief to individuals with anxiety related to asthma. The data from this study provide the basis for the current randomised controlled trial which will be conducted to evaluate efficacy.

### Aim and objectives

The overall aim of this current study is to test Internet-CBT as a treatment for individuals experiencing anxiety related to asthma. The specific research questions are the following:

1. Is Internet-CBT more effective than treatment as usual+medical education (TAU+ME) to improve asthma-related anxiety, asthma control, FAS, excessive avoidance behaviours, perceived stress, worry, anxiety sensitivity, health anxiety, insomnia, mood and quality of life in individuals with anxiety related to asthma?
2. Is Internet-CBT more effective than TAU+ME in improving objective measures of asthma control (physical lung function) in people with anxiety-related asthma?

## Methods and analysis

This will be a two-arm, parallel-group randomised controlled trial to investigate the superiority of Internet-CBT compared with TAU. 90 participants will be nationally recruited from advertisements in social media and newspapers, and randomised in a 1:1 ratio. Due to the nature of the intervention and the early phase of evaluation with a TAU group, participants will not be blinded to group allocation (see the statistical analysis plan for further details (online supplemental file 1)).

10.1136/bmjresp-2023-002035.supp1 Supplementary data

### Study setting

All study procedures will be carried out online via the internet. All participants will be residing in Sweden, and all written materials will be in Swedish.

### Eligibility criteria

#### Inclusion criteria

1. Self-reported asthma as diagnosed by a physician.
2. Self-reported anxiety or worry related to asthma (ie, asthma leads to significant distress or interferes with daily life).
3. Age >18 years.
4. Daily access to the internet and some computer skills.

#### Exclusion criteria

1. Newly introduced or dose-adjusted psychotropic medication (in the last 2 months).
2. Severe psychiatric illness, for example, suicidality, drug or alcohol abuse or psychotic disease.
3. Severe respiratory disease, other than asthma (ie, as diagnosed by a physician or a history of smoking at least 1 pack of cigarettes daily for 10 years (10 pack-years) which increases the risk for COPD).
4. Ongoing structured psychological treatment (ie, manual-based CBT or interpersonal psychotherapy).
5. Severe cognitive impairment or significant difficulties in reading and writing Swedish.

### Intervention

#### Internet-CBT

The treatment comprises 8-weekly written modules delivered over the internet. The outline is based on a fear-avoidance model which puts a strong emphasis on hypersensitivity and attentional bias to asthma symptoms.^34^ The treatment consists of written texts and case examples to teach about asthma and anxiety and the main strategy, exposure to situations that may elicit fear of asthma. The gradual exposure to asthma-related anxiety could, for example, consist of increased physical activity such as taking the stairs instead of the lift or speaking in front of others despite symptoms of asthma. Prior to the exposure phase, the importance of having regular asthma medication is emphasised, and that exposure exercises should not include obvious asthma triggers such as smoke or allergens. Affective labelling is taught as a strategy to reduce negative emotional reactivity to asthma symptoms and thus be able to facilitate exposure to the fearful stimuli. Because some have disturbed sleep due to asthma, sleep hygiene is included in one module. The last module includes a summary of taught strategies and a plan for maintaining behavioural change (see table 1 for outline of the treatment).

**Table 1:** Treatment outline for the 8-weekly modules of the Internet-CBT

	Education about asthma and anxiety	Medical adherence	Affective labelling	Exposure	Sleep	Maintain behavioural change
Week 1	X	X*		X		
Week 2			X	X		
Week 3				X		
Week 4		X	X	X		
Week 5				X	X	
Week 6				X		
Week 7			X	X		
Week 8				X		X

*The module about medical adherence is also distributed to the TAU+ME group at week 1.

CBT, cognitive–behavioural therapy; TAU+ME, treatment as usual+medical education.

Throughout the treatment, participants have regular contact with an assigned therapist through a secure web platform, where also the treatment content is presented. Participants are encouraged to send weekly reports about their homework assignments to their therapist via the platform and the therapist provides feedback within two working days via text-messages online. Participants can also contact their therapist through messaging within the platform with questions during the treatment, and therapists may remind participants that lag to login, primarily through text messages from the platform and by phone only when a participant has not answered their weekly assignment for 5 days. All therapist contact is handled by licensed psychologists trained in CBT.

#### Treatment as usual+medical education

The ME about asthma and a clear call to follow prescribed asthma medication is distributed through one online module in the platform to the control group. This information is identical to what the Internet-CBT group receives and aims to control for possible effects of increased medical adherence, that is, it is not only the information about medication and the call to follow prescribed medication that produces an effect (if an effect is observed after Internet CBT). The TAU+ME group does not have any therapist support.

Both groups can use any other concurrent treatment available during this study phase, besides ongoing psychotherapy.

### Outcome measures

All assessments are conducted over the internet without any influence from study staff.

#### Assessment points

Two weeks prior to randomisation, weekly for 8 weeks postrandomisation, and then at week 16 postrandomisation (see table in online supplemental file 2 for details).

10.1136/bmjresp-2023-002035.supp2 Supplementary data

#### Primary outcome measure

CAS is a validated scale to assess catastrophising thoughts about asthma. The scale consists of 21 items, including 10 items on catastrophising during an asthma attack and 11 items measuring CAS in daily life.^35^

#### Secondary outcome measures

1. The Asthma Control Test (ACT) is a five-item questionnaire to assess perceived symptom severity. ACT has shown good reliability and discriminant validity.^36 37^
2. The Asthma Behaviour Checklist (ABC) is developed by the research group to assess asthma-related behavioural avoidance and consists of items such as: ‘(Due to my asthma) I often check my breathing’, ‘(Due to my asthma) I avoid taking walks with other people’. The ABC comprise eight statements on a seven-graded scale, from 0 (never) to 7 (always).^38^
3. FAS is a validated scale assessing symptomatic sensitivity related to asthma and includes items such as ‘Whenever I feel symptoms of asthma, I become anxious’ and ‘I’m always aware of my breathing’.^38^ FAS consists of 11 items on a scale from 0 (do not agree at all) to 5 (very much agree).
4. Perceived Stress Scale (PSS-10): PSS-10 is an often-used instrument to assess an individual’s experienced stress.^39 40^
5. The Penn State Worry Questionnaire (PSWQ) is a self-report measure comprising 16 items, with each item rated on a 5-point scale.^41^ The PSWQ is a well-validated instrument, that is, recommended when assessing the process of worry.^42^
6. Anxiety Sensitivity Index (ASI) is a validated instrument to assess fear of anxiety symptoms and comprises 16 items.^43^
7. Short Health Anxiety Inventory (SHAI) assesses concern for health, awareness of bodily sensations and changes, and fear of the consequences of disease. SHAI has demonstrated good reliability and validity in both clinical and healthy groups.^44 45^
8. Insomnia Severity Index contains seven items and is an often-used validated scale to assess sleeping problems.^46^
9. The Brunnsviken Brief Quality of Life Scale assesses general life quality and is validated in Sweden.^47^
10. To assess depression, the Patient Health Questionnaire-9 (PHQ-9) will be used as a screening instrument and as a secondary outcome.^48^
11. Participants will use an app to measure lung function, forced expiratory volume in 1 s (AsthmaTuner).^49^ The lung function measurements will be conducted twice daily for 10 days at baseline, post-treatment and at 16 weeks follow-up.
  Process outcomes:
12. Client Satisfaction Questionnaire-8 is an 8-item scale that assesses satisfaction with the treatment, the scale has commonly been used in treatment trials evaluating CBT.^50^
13. Adverse events comprise a short scale asking participants about any possible unwanted events during therapy, with the purpose to systematically collecting data on any adverse events due to CBT.^51^
14. Subjective Assessment Questionnaire uses one item to assess participants’ perceived global symptom improvement or deterioration due to treatment.^52^
15. Working Alliance Inventory is a 6-item questionnaire administered during the treatment to assess the participants perceived alliance with the therapist.^53 54^

### Sample size and power

A power analysis using G*power demonstrated that the sample size of 90 will provide 80% power at alpha-level 0.05 to detect a moderate between-group effect size (Cohen’s d=0.6) on the primary outcome (catastrophising about asthma) from prerandomisation to 16 weeks postrandomisation. The analysis is based on the treatment effects in the feasibility study. We assumed an SD of 1.0.

### Recruitment

Participants will be nationally recruited from advertisements and apply online by completing screening instruments and leaving digital consent to study participation. Eligible participants with a complete application will receive a clinical psychological assessment by phone to exclude serious psychiatric problems that require immediate psychiatric care (eg, psychosis, addiction problems or severe depression) or that obviously make it difficult for the participant to benefit from the treatment (eg, severe cognitive impairment or language difficulties). Participants who meet any exclusion criteria at the screening or during the interview will be contacted by phone and if needed referred to other care. At the clinical interview, an experienced clinical psychologist will ensure that the participant have read and understood the study information and still leave consent to participate in the study. Participants who are judged eligible will be randomised after completing all preassessments.

#### Screening measures

The PHQ-9 is a well-used nine-item depression scale designed to screen for severity of depression. Alcohol use disorders identification test and drug use disorders identification test are well-used instruments to identify substance abuse.^40–43^ During the screening, participants fill out a demographic questionnaire, questions about their asthma, current and previous somatic and psychiatric diagnoses, as well as questions about smoking. The Mini-International Neuropsychiatric Interview (M.I.N.I.) is a short, structured interview to screen for psychiatric ICD-10 or Diagnostic and Statistical Manual of Mental Disorders, 4th Edition (DSM IV) diagnoses, developed for use in clinical trials and epidemiological studies.^44^ M.I.N.I. will be assessed during the clinical interview.

### Randomisation

Participants will be randomised in a 1:1 ratio to Internet-CBT or to TAU+ME. Participants randomised to TAU+ME will be offered the Internet-CBT with therapist support after the primary endpoint, 16 weeks after randomisation. After the cross-over, data from the comparison group is no longer used in the study. The randomisation will be conducted by an external researcher. Study staff will not be concealed in the allocation of participants.

### Data collection

All data will be collected online using the BASS platform and server that is part of the core facilities at Karolinska Institutet, Stockholm, Sweden. Automatic reminders will be sent from the platform to the participants when an assessment is due. Thus, study staff will have no influence on data collection.

### Statistical analyses

The main statistical analyses in the randomised controlled trial will be conducted according to the ‘intention-to-treat’ principle, including all participants in the arm to which they were randomised. The primary analysis will be a hierarchical linear mixed model of the slope over time, up to 16 weeks after randomisation. More details are given in the statistical analysis plan (online supplemental file 1) and in the shell tables (online supplemental file 3).

10.1136/bmjresp-2023-002035.supp3 Supplementary data

Because previous studies have not shown any risk with CBT for this population, there are no preplanned interim analysis or predefined stopping rules.

## Patient and public involvement

During treatment development, the participants in the prior studies have been given the opportunity to provide feedback on treatment components.^33 34^ Received feedback influenced further treatment development, such as clarifying examples or shortening of text sections. Through user participation from the Swedish Asthma and Allergy Association, treatment protocol and measurement batteries were reviewed to ensure they aligned well with the needs and expectations of the target group.

## Clinical relevance

This research project may lead to an effective treatment available for the many struggling with asthma complicated by anxiety, who currently do not have access to evidence-based treatments.

## Dissemination

Internet-based CBT differs from traditional CBT mainly through how the therapist’s contact is managed. Any serious adverse effects of this type of treatment have not been reported in the literature, nor can we predict any obvious risks due to participation in the treatment or assessment procedure. To ensure that this is the case also in this study, data on any adverse events will be collected. Since the participant is in regular contact with their therapist, there are very good opportunities to detect acute problems that may arise. Normal patient injury compensation applies to study participants in this study. The communication between participant and therapist is protected by secure cryptographic certificates and all authentication is protected by using both username/password and a temporary password sent by SMS (double authentication). All participants will be informed about and give their consent for digital storage of personal data in accordance with General Data Protection Regulation (GDPR; see online supplemental file 4 for the informed consent). The results of the study will be analysed at the group level and reported through publication in a peer-reviewed scientific journal within the field. Authorship will follow the International Committee of Medical Journal Editords (ICJME) 2018 guidelines. Due to Swedish data storage laws, we cannot make the data publicly available. However, pseudonymised data may be provided by the PI on requests if providing a reasonable proposal and if an appropriate data sharing agreement with Karolinska Institutet can be established.

10.1136/bmjresp-2023-002035.supp4 Supplementary data

10.1136/bmjresp-2023-002035.supp5 Supplementary data

## Data Availability

No data are available. Due to Swedish data storage laws, we cannot make the data publicly available. However, pseudonymised data may be provided on requests if providing a reasonable proposal and if an appropriate data sharing agreement with Karolinska Institutet can be established.
