# Supplementary material for: Internet-delivered cognitive–behaviour therapy for anxiety related to asthma: study protocol for a randomised controlled trial
Source: BMJ Open Respir Res. 2024 May 27;11(1):e002035. doi: 10.1136/bmjresp-2023-002035 (PMC11131118; doi:10.1136/bmjresp-2023-002035)
Supplement: Supplementary data [file bmjresp-2023-002035supp001.pdf]

Supplement to Internet-delivered Cognitive Behavior Therapy for Anxiety Related to Asthma: Study Protocol for a Randomized Controlled Trial

SUPPLEMENT 1: STATISTICAL ANALYSIS PLAN

Objectives

(1) Can Internet-CBT be more effective than Treatment as Usual + Medical Education (TAU+ME) to improve: (a) asthma anxiety (CAS), (b) asthma control (ACT), (c) avoidance behavior (ABC), (d) fear of asthma symptoms (FAS), (e) perceived stress (PSS), (f) worry (PSWQ), (g) anxiety sensitivity (ASI), (h) health anxiety (SHAI), (i) insomnia (ISI), (j) depression (PHQ-9) and (k) quality of life (BBQ) in people with anxiety-related asthma?

(2) Can Internet-CBT be more effective than TAU+ME in improving objective measures of asthma control (Forced expiratory volume, FEV1) in people with anxiety-related asthma?

STUDY METHODS

Trial design

This is a randomized, two-arm parallel controlled trial. Consenting participants will be randomized 1:1 to receive eight weeks of either internet-CBT (the intervention) or TAU+ME (the control). The primary outcome measure is each particpant's score on the Catastrophizing about asthma Scale (CAS). This will be recorded at baseline (two weeks before start of therapy) and each week during the therapy (weeks 1 to 8), and again at week 16 week.

Randomization

Participants will be consecutively 1:1 randomized after pre-assessments. The randomizer is a researcher outside of the research project who recieves study codes and uses the online list randomizer at random.org to randomize the order of participants and to create a randomized list of the two conditions, Internet-CBT or TAU+ME. The participant will then receive the condition paired to the order (study code randomized to order 1 will receive the condition randomized to order 1, study code randomized to order 2 the condition randomized to order 2 etc.). All randomized sets are timestamped and the results are saved by the external randomizer.

Sample size

We aim to recruit 90 participants in total. A power analysis using G*power demonstrated that this will provide 80% power at alpha-level 0.05 to detect an effect of Cohen´s *d*=0.6 on the CAS score from pre-randomisation to 16 weeks post-randomisation. This is a conservative estimate compared to the effect we observed in the pilot study (*d*=1.5). We assumed a standard deviation of 1.0.

Framework

All comparisons will be made on a two-sided superiority basis. The primary comparison is of average slope of CAS during the trial, compared between the two randomisation groups on an intention-to-treat basis.

Statistical interim analysis and stopping guidance

Supplement to Internet-delivered Cognitive Behavior Therapy for Anxiety Related to Asthma: Study Protocol for a Randomized Controlled Trial

There are no plans for interim analyses or checkpoints for stopping the trial. Internet-CBT is a well established method of delivering therapy, and we consider the potential for harm very low.

Timing of final analysis

The final analysis will be conducted 16 weeks after the last participant has been randomized, ie after all participants are scheculed to have completed the primary endpoint assessment.

STATISTICAL PRINCIPLES

Confidence intervals and p-values

We will use a significance level of 5% throughout, and report corresponding symetrical 95% confidence intervals.

Adherence and Protocol deviations

Participants who complete at least 4 weeks of Internet-CBT will be defined as completers as they have received the main components of the treatment at that time. Any other treatment received during the study period will be recorded in both groups using online self-assessment questionnaires.

Analysis population

We will use just one study population, the Intention-to-treat (ITT) population. This will consist of all randomized participants, who will be analyzed according to their original assignment. All participants with at least a baseline measure and one subsequent measure of CAS will be included.

TRIAL POPULATION

Screening data

All participants that have signed up to the study have given digital informed consent to use initial screening data, after which they have answered the online-screening collected through digital assessments using a secure platform (BASS) run by and stored at Karolinska Institutet, Stockholm, Sweden. Hence, data on both included and excluded participants will be reported, including demographics, self-reported comorbid psychiatric disorders and somatic diseases as well as data on duration of asthma diagnosis, medication and smoking history.

Eligibility

Inclusion criteria:
(1) Self-report of asthma as diagnosed by a physician;
(2) Self-reported anxiety or worry related to asthma (i.e., asthma leads to significant distress or interferes with daily life);
(3) Age ≥18 years;
(4) Daily access to the internet and some computer skills.

Exclusion criteria:
(1) Newly introduced or dose-adjusted psychotropic medication (in the last 2 months);

Bonnert M, *et al. BMJ Open Respir Res* 2024; 11:e002035. doi: 10.1136/bmjresp-2023-002035

Supplement to Internet-delivered Cognitive Behavior Therapy for Anxiety Related to Asthma: Study Protocol for a Randomized Controlled Trial

(2) Severe psychiatric illness e.g., suicidality, drug- or alcohol abuse or psychotic disease;
(3) Severe respiratory disease, other than asthma (i.e., as diagnosed by a physician or a history of smoking at least 10 pack-years which increase the risk for COPD);
(4) Ongoing structured psychological treatment (i.e., manual-based CBT or Interpersonal psychotherapy);
(5) Severe cognitive impairment or significant difficulties in reading and writing Swedish.

Recruitment
We will complete a CONSORT flow diagram to illustrate participant flow through the trial.

Withdrawal / Follow-up
We will complete Shell Table 1 (see supplement 3)

Baseline patient characteristics
We will complete Shell Table 2 (see supplement 3).

ANALYSIS
Outcome definitions

Primary outcome
The primary outcome is each individual's total score on the Catastrophizing about Asthma Scale (CAS; [1]). This is a validated scale to assess catastrophizing thoughts about asthma, which is a construct close to anxiety related to asthma. The scale consists of 24 items, including 10 items on catastrophizing during an asthma attack, and 11 items measuring catastrophizing about asthma in daily life. The CAS is calculated by summing the responses to 24 questions, each with a possible whole number score from zero to four. Hence the possible values are whole numbers from zero to 96.

Secondary outcomes
1. The two subscales of the CAS:
   (a) **CAS Exacerbation**: 13 items, maximum score of 52
   (b) **CAS General**: 11 items, maximum score of 44
2. **Asthma control test (ACT)**. A five-item questionnaire, the range of scores for each question is 1-5, hence the range for the total is 5-25 [2,3]
3. **Asthma Behavior Checklist (ABC).** An 8 item scale, each response is scored fom 0 to 7, so the total range is 0-56 [4]
4. **Fear of Asthma Symptoms (FAS)**.This is a 11-item scale, with all questions scored from zero to four. The range for the total score is thus 0-44 [4]
5. **Perceived Stress Scale (PSS)**. A 10-item questionnaire, with responses scored from 0-4. Thus the total range is 0-40 [5,6]
6. **Penn State Worry Questionnaire (PSWQ)**. A 16 item scale, with each item rated on a 1-5 point scale. The total range is thus 16-80 [7].
7. **Anxiety Sensitivity Index-3 (ASI)**. An 16-item measure, with each item rated on a Likert scale of 0-4. Thus the total range is 0-64 [8].
8. **Short Health Anxiety Inventory (SHAI)**. This is an 18-item scale with each item rated on a 0-3 scale and a total range of 0-54 [9].

Bonnert M, et al. BMJ Open Respir Res 2024; 11:e002035. doi: 10.1136/bmjresp-2023-002035

Supplement to Internet-delivered Cognitive Behavior Therapy for Anxiety Related to Asthma: Study Protocol for a Randomized Controlled Trial

9. **Insomnia Sleep Inventory (ISI).** The scale comprise 7 items rated on a 0-4 scale, total rangre 0-28 [10].
10. **Brunnsviken Brief Quality of Life Scale (BBQ)**. A 12 question scale across six areas. Each question is a rated on a five-step Likert scale, scored 0–4. The total is calculated by multiplying the weight in each area by that area's weight. The possible scoring range is 0-96 [11].
11. **Patient health questionnaire (PHQ-9).** This is a 9-item questionnaire, with each item scored 0-3. The total possible range thus is 0-27[12].
12. **Forced expiratory volume per second (FEV1).** FEV1 will be assessed with the digital spirometer AsthmaTuner® [13]. The lung function measurements will be conducted twice daily during ten days at baseline, posttreatment and at 2 months follow-up. Hence, participants may contribute 10-60 assessments of FEV1. All possible assessment points will be included in the ITT analysis.

Analysis methods
For the primary outcome, we will use the CAS score from all timepoints (at baseline, weekly during treatment, and at week 16) in a hierarchical model, with a random intercept for each individual. This assumes a normal distribution for the deviations from the mean at each timepoint. We will include an interaction between treatment and slope of CAS: the primary hypothesis is that this interaction coeffecient is not equal to zero. We will use the likelihood ratio test of this interaction term to test this hypothesis. A negative slope indicates an improvement in catostrophizing symptoms, hence we aim to show that the slope parameter for participants in the intervention arm is lower than the slope parameter in the TAU+ME arm. We do not plan to include any covariates in the model, other than the random assignation. However, if we observe an unexpectedly large difference in any of the factors in Shell Table 2 we may include that variable.

In addition, we will report the change in CAS at the 16 week timepoint, adjusted for the baseline score using a linear regression model (fixed effects only) including just the CAS score at baseline, the change in CAS at 16-week follow-up, the randomized assignation, and any potential confounders as identified above.

We will report the results from the primary analysis in Shell Table 3 (see supplement 3).

Secondary outcomes will be assessed using the same methods as described above. We will report the results from the secondary analyses in Shell Table 4 (see supplement 3).

Effect-sizes will be estimated based on the standardized mean difference (Cohens's *d*) between groups at week 16 in primary and secondary outcomes. Numbers needed to treat (NNT) will be based on the Cohen´s d estimation [14].

Missing data
All randomized participants will be included in the analysis. Imputation may be used if data attrition is higher than 10% using bootstrap estimation with multiple imputation.

Supplement to Internet-delivered Cognitive Behavior Therapy for Anxiety Related to Asthma: Study Protocol for a Randomized Controlled Trial

Additional analyses

An exploratory analysis will be conducted to investigate the dose-response of Internet-CBT on CAS. This analysis will use the number of completed treatment modules as a continuous exposure variable, which can take values from zero to eight. Individuals randomized to the TAU+ME group will be set to 0 as they will not have received any modules. We will use a linear mixed effect model (with a random intercept for each individual) to estimate the average change in CAS score per completed module.

Harms

Data on any adverse events during the treatment will be collected at post-assessments.

Statistical software

Analysis will be performed in Stata, version 17 or higher.

References

1 Peuter SD, Victoir A, Lemaigre V, *et al.* Catastrophic thinking and symptom perception in asthma: Validation of a questionnaire. *The Internet Journal of Asthma, Allergy and Immunology* 2006;**5**. doi:10.5580/8fd

2 Nathan RA, Sorkness CA, Kosinski M, *et al.* Development of the asthma control test: a survey for assessing asthma control. *J Allergy Clin Immunol* 2004;**113**:59–65. doi:10.1016/j.jaci.2003.09.008

3 Schatz M, Sorkness CA, Li JT, *et al.* Asthma Control Test: reliability, validity, and responsiveness in patients not previously followed by asthma specialists. *J Allergy Clin Immunol* 2006;**117**:549–56. doi:10.1016/j.jaci.2006.01.011

4 Bonnert M, Roelstraete B, Bergstrom S-E, *et al.* The Fear of Asthma Symptoms Scale and the Asthma Behavior Checklist: preliminary validity of two novel patient reported outcome measures. *J Asthma* 2022;:1–15. doi:10.1080/02770903.2022.2160343

5 Cohen S. Perceived stress in a probability sample of the United States. Published Online First: 1988.https://psycnet.apa.org/record/1988-98838-002

6 Nordin M, Nordin S. Psychometric evaluation and normative data of the Swedish version of the 10-item perceived stress scale. *Scand J Psychol* 2013;**54**:502–7. doi:10.1111/sjop.12071

7 Meyer TJ, Miller ML, Metzger RL, *et al.* Development and validation of the penn state worry questionnaire. *BRAT* Published Online First: 1990. doi:10.1016/0005-7967(90)90135-6

8 Reiss S, Peterson RA, Gursky DM, *et al.* Anxiety sensitivity, anxiety frequency and the prediction of fearfulness. *Behav Res Ther* 1986;**24**:1–8. doi:10.1016/0005-7967(86)90143-9

9 Salkovskis PM, Rimes KA, Warwick HMC, *et al.* The Health Anxiety Inventory: development and validation of scales for the measurement of health anxiety and hypochondriasis. *Psychol Med* 2002;**32**:843–53. doi:10.1017/s0033291702005822

Supplement to Internet-delivered Cognitive Behavior Therapy for Anxiety Related to Asthma: Study Protocol for a Randomized Controlled Trial

10 Bastien C, Vallières A, Morin CM. Validation of the Insomnia Severity Index as an outcome measure for insomnia research. *Sleep Medicine* 2001;**2**:297–307. doi:10.1016/s1389-9457(00)00065-4

11 Lindner P, Frykheden O, Forsström D, *et al.* The Brunnsviken Brief Quality of Life Scale (BBQ): Development and Psychometric Evaluation. *Cogn Behav Ther* 2016;**45**:182–95. doi:10.1080/16506073.2016.1143526

12 Kroenke K, Spitzer RL, Williams JB. The PHQ-9: validity of a brief depression severity measure. *J Gen Intern Med* 2001;**16**:606–13. doi:10.1046/j.1525-1497.2001.016009606.x

13 Ljungberg H, Carleborg A, Gerber H, *et al.* Clinical effect on uncontrolled asthma using a novel digital automated self-management solution: a physician-blinded randomised controlled crossover trial. *Eur Respir J* 2019;**54**. doi:10.1183/13993003.00983-2019

14 Preti A. How to calculate the Number Needed to Treat (NNT) from Cohen's d or Hedges'g. 2015.https://rpubs.com/RatherBit (accessed 2015).
