# Supplementary material for: Internet-delivered cognitive–behaviour therapy for anxiety related to asthma: study protocol for a randomised controlled trial
Source: BMJ Open Respir Res. 2024 May 27;11(1):e002035. doi: 10.1136/bmjresp-2023-002035 (PMC11131118; doi:10.1136/bmjresp-2023-002035)
Supplement: Supplementary data [file bmjresp-2023-002035supp002.pdf]

Supplement to Internet-delivered Cognitive Behavior Therapy for Anxiety Related to Asthma: Study Protocol for a Randomized Controlled Trial

Supplement 2. Schedule of enrolment and outcome assessment points

				STUDY PERIOD									
	Enrolment	Pre-ass.	Allocation	ICBT or TAU+ME								Primary endpoint	FU (ICBT)
**TIMEPOINT (Week)**	*-3*	*-2*	*0*	*1*	*2*	*3*	*4*	*5*	*6*	*7*	*8*	*16*	*32*
**Eligibility screen**	X												
**Informed consent**	X												
Pre-assessments + AstmaTuner (twice daily x 10 days)		X											
**1:1 randomization**			X										
**OUTCOME ASSESSMENTS**													
**Primary outcome: *Asthma-anxiety, CAS***		X		X	X	X	X	X	X	X	X	X	X
**Secondary Outcomes: *Asthma symptoms, ACT***		X		X	X	X	X	X	X	X	X	X	X
Fear of asthma symptoms, FAS		X		X	X	X	X	X	X	X	X	X	X
Excessive avoidance, ABC		X		X	X	X	X	X	X	X	X	X	X
Perceived stress, PSS-10		X		X	X	X	X	X	X	X	X	X	X
Worry, PSWQ		X									X	X	X

Note. The table continues in next page.

BMJ Publishing Group Limited (BMJ) disclaims all liability and responsibility arising from any reliance placed on this supplemental material which has been supplied by the author(s)

Supplement to Internet-delivered Cognitive Behavior Therapy for Anxiety Related to Asthma: Study Protocol for a Randomized Controlled Trial

S2. Continued

				**STUDY PERIOD**									
	**Enrolment**	**Pre-ass.**	**Allocation**	**ICBT or TAU+ME**								**Primary endpoint**	**FU (ICBT)**
**TIMEPOINT (Week)**	***-3***	***-2***	***0***	***1***	***2***	***3***	***4***	***5***	***6***	***7***	***8 post-ass.***	***16***	***32***
Anxiety Sensitivity, ASI		**X**									**X**	**X**	**X**
Health Anxiety, SHAI		**X**									**X**	**X**	**X**
Insomnia, ISI		**X**									**X**	**X**	**X**
Depression, PHQ-9		**X**									**X**	**X**	**X**
Quality of life, BBQ		**X**									**X**	**X**	**X**
FEV1, AsthmaTuner		**X**									**X**	**X**	

Note. ICBT=internet-delivered cognitive behavior therapy; TAU+ME =treatment as usual plus medical education; Pre-ass=assessments before intervention; CAS=Catastrophizing about Asthma Scale; ACT=Asthma Control Test; FAS=Fear of Asthma Symptoms scale; ABC=Asthma Behavior Checklist; PSS=Perceived Stress Scale; PSWQ=Penny State Worry Questionnaire; ASI=Anxiety Sensitivity Index; SHAI=Short Health Anxiety Inventory; ISI=Insomnia Severity Index; PHQ= Patient Health Questionnaire; BBQ= Brunnsviken Brief Quality of Life Scale; FEV1=Forced expiratory volume per 1 second. AsthmaTuner is a registered digital spirometer which participants connect to an App and use at home.
