# Supplementary material for: Internet-delivered cognitive–behaviour therapy for anxiety related to asthma: study protocol for a randomised controlled trial
Source: BMJ Open Respir Res. 2024 May 27;11(1):e002035. doi: 10.1136/bmjresp-2023-002035 (PMC11131118; doi:10.1136/bmjresp-2023-002035)
Supplement: Supplementary data [file bmjresp-2023-002035supp003.pdf]

Supplement to Internet-delivered Cognitive Behavior Therapy for Anxiety Related to Asthma: Study Protocol for a Randomized Controlled Trial

Supplement 3: Shell tables

Shell Table 1

Description	Internet-CBT arm (N=xx)	TAU+ME arm (N=xx)
Completed all planned modules and outcome measures	xx (xx.x%)	xx (xx.x%)
Completed at least four modules of ICBT/1 module of TAU	xx (xx.x%)	xx (xx.x%)
Number of modules taken: median (IQR)	xxx (xx – xx)	xxx (xx – xx)
Number of CAS measures: median (IQR)	xxx (xx – xx)	xxx (xx – xx)
Completed assessment at the primary endpoint	xx (xx.x%)	xx (xx.x%)

Shell Table 1. Withdrawals and adherence to study procedures after randomization

Shell Table 2

Characteristic	Internet-CBT arm (N=xx)	TAU+ME arm (N=xx)
Sex: number female (%)	xx (xx)	xx (xx)
Age: median (IQR)	xx (xx – xx)	xx (xx – xx)
Education		
Asthma duration		
Asthma Severity		
Asthma Medication		
Allergy		
Psychiatric disorders		
Psychotropic medicine		
Other important characteristics		

Shell Table 2. Baseline characteristics of participants

Shell Table 3

Measure	Internet-CBT arm (N=xx)	TAU+ME arm (N=xx)	Difference (Internet-CBT – TAU)	95% CI	p-value	Effect size Cohens´d [CI]
Baseline score, mean (SD)	xx.xx	xx.xx	xx.xx	(xx.x, xx.x)	---	
16-week score: mean (SD)	xx.xx	xx.xx	xx.xx	(xx.x, xx.x)	---	x.xx [x.xx, x.xx]
Slope parameter (weekly change) *	xx.xx	xx.xx	xx.xx	(xx.x, xx.x)	0.xxx	

Shell table 3. Results of primary analysis (CAS)

* from multi-level regression model, with random intercept for each participant

Supplement to Internet-delivered Cognitive Behavior Therapy for Anxiety Related to Asthma: Study Protocol for a Randomized Controlled Trial

Shell Table 4

Measure	Baseline: mean (SD)	16-week: mean (SD)	Difference in slopes (Internet-CBT vs TAU+ME): mean (SD)	95% CI	p-value	Effect size Cohens´d [CI]
CAS Exacerbation	xx.xx (xx.xx)	xx.xx (xx.xx)	xx.xx (xx.xx)	(xx.x, xx.x)	0.xxx	x.xx [x.xx, x.xx]
CAS General						
Asthma control test						
Asthma Behavior Checklist						
Fear of Asthma Symptoms						
Perceived Stress Scale						
Penn State Worry Questionnaire						
Anxiety Sensitivity Index-3						
Short Health Anxiety Inventory						
Insomnia Sleep Inventory						
Brunnsviken Brief Quality of Life Scale						
Depression, PHQ-9						
FEV1						

Shell table 4. Results of secondary analyses
