# Supplementary material for: Internet-delivered cognitive–behaviour therapy for anxiety related to asthma: study protocol for a randomised controlled trial
Source: BMJ Open Respir Res. 2024 May 27;11(1):e002035. doi: 10.1136/bmjresp-2023-002035 (PMC11131118; doi:10.1136/bmjresp-2023-002035)
Supplement: Supplementary data [file bmjresp-2023-002035supp004.pdf]

SUPPLEMENT 4: Participants digital consent translated from Swedish.

Research Participant Information and Consent

Request for participation in a study on Internet-CBT for asthma and anxiety.

Background

The association between asthma and anxiety or stress is well-established. Individuals with anxiety and stress are at a greater risk of developing asthma, and anxiety in asthma is related to impaired asthma control and decreased quality of life. Cognitive Behavioral Therapy (CBT) has shown promise in improving the quality of life, asthma control, and anxiety for adults with asthma. Internet-delivered Cognitive Behavioral Therapy (Internet-CBT) with therapist support has proven to be an effective treatment for anxiety and depression, with equivalent effects to traditional CBT. Internet-CBT follows the same principles as traditional CBT, with the main difference being that the treatment is delivered over the internet with therapist support. We have previously investigated whether Internet-CBT can reduce anxiety and improve asthma control. The results are very promising, and participants were satisfied with the treatment. Hence, we now want to investigate whether Internet-CBT may work better than standard care alone.

Target Group

The target group for our study is individuals over 18 years old with asthma and anxiety, who feel anxious about their asthma or perceive that asthma significantly impacts their daily lives.

Study Procedure

You read through this entire information and then decide whether you want to participate or not by clicking on "I consent" at the bottom of the page. By doing so, you also consent to us accessing previous health care records, and your medical data will be linked to national health and quality registers for additional information on outcomes and long-term follow-up. The information collected pertains to the care you receive for asthma and anxiety, as well as any impact on work capacity. The data will be analyzed at a group level, and no presented data can be linked to individual participants. If you have questions about the study, you can contact the study's contact person, Marianne Bonnert, by phone or email (see below).

If you agree to participate in the study, you will be immediately directed to the screening process. The screening consists of questions about your health, anxiety, and asthma. We also inquire about your occupation and functional level. It takes about 15 minutes to answer the questions.

After completing the screening, we will contact you to schedule a time for an assessment interview over the phone. If the screening indicates that study participation is not applicable, you will be informed, and if necessary, referred to another treatment. The assessment interview takes about an hour and is conducted by a licensed psychologist or psychology student under the supervision of a licensed psychologist. The purpose of the call is to assess if treatment is suitable for you. The interview explores any past or ongoing psychiatric issues. You will have the opportunity to ask questions about your participation in the study, and we will ask if you are still interested in participating. Even if you consent to participate, you have the right to withdraw at any time without providing an explanation.

SUPPLEMENT 4: Participants digital consent translated from Swedish.

After the assessment interview, you will answer new questions online in a pre-measurement. You will also receive a simple device measuring your lung function (spirometer) called Asthma Tuner, along with instructions on usage.
The Asthma Tuner is connected to an app that you download to your smartphone. You will measure your lung function using the Asthma Tuner before and after treatment, as well as after 2 months. You are expected to blow into the Asthma Tuner twice daily for 2 weeks at each measurement session.

Randomization
To compare whether Internet-CBT is better than standard care, we need to follow individuals receiving the treatment and compare them with those not receiving it over a period. This means that all participants will be randomly assigned to either receive the treatment immediately or after 4 months. This is called randomization. After completing the pre-measurement and using your Asthma Tuner for 2 weeks, you will be randomized to either immediate treatment or to continue as usual for 4 months before receiving the treatment.

Internet-CBT
The Internet-CBT will last for 8 weeks with a new module each week. You work independently with the program from home and will receive exercises to be performed during the week, some of which are done daily. It is crucial to complete the exercises for the treatment to be effective. Each module contains information and a presentation of exercises or tasks. You will receive feedback from your therapist via the internet program and you can ask questions to the therapist throughout the treatment.

Standard Care
If you are randomized to continue as usual for 4 months, you will receive Internet-CBT after the 4 months. During this time, you continue to access and receive the care available to you. You will also receive internet-delivered information about asthma and access to a medication plan that you can use. For 8 weeks, you will answer questions about your well-being once a week. In the eighth week, you will answer additional questions in what we call a post-measurement, and you will also use the Asthma Tuner again. After that, there is a break. After two months, we will contact you again, and it is time for you to answer a new set of questions and blow into the Asthma Tuner. Once that is completed, you will be offered to start Internet-CBT.

Handling of Data and Privacy
All data collected will only be accessed by personnel in the research group, who have complete confidentiality, and all journal documents are subject to confidentiality and privacy protection. Our study is designed according to the rules for personal data in research found in the EU's General Data Protection Regulation. Your responses and results will be processed to prevent unauthorized access. The database used in the treatment and for collecting responses is specifically designed for gathering research data in internet-based treatment and is protected by encryption and double-authentication or login with BankID.

How Do We Protect Your Personal Data?

SUPPLEMENT 4: Participants digital consent translated from Swedish.

Personal data is processed in accordance with the European General Data Protection Regulation 2016/679 (GDPR). The personal data stored includes your contact information (postal address, email address, and your mobile number) and your personal identification number. Personal data is stored in a separate location after being assigned a code number. Data is then analyzed in coded form and stored for at least 10 years according to applicable rules, after which the code key is destroyed. Data may be used in future research after new ethical review. The data controller is Karolinska Institutet, and by contacting dataskyddsombud@ki.se, you can request an extract of the personal data stored about you or request corrections to any incorrect personal data. If you are dissatisfied with how Karolinska Institutet handles your personal data, you can contact the Swedish Data Protection Authority with complaints via email: datainspektionen@datainspektionen.se.

What Does the Study Entail?
Participating in the study involves no specific risks. If you have questions or if your participation raises thoughts you would like to discuss with someone, you can contact the study's contact person (below) or contact primary care. Participation in the study is entirely voluntary. You can withdraw your participation at any time without any negative consequences.

Results
The results of the study will be compiled into articles and presented in scientific journals, as well as at conferences. The data you contribute may be the basis for publication in Swedish and/or international journals, mainly on a group level and always completely anonymized. No one will be able to connect any answers to any specific person.

Insurance and Compensation

Normal patient injury compensation applies to study participants in this study. The treatment is free of charge. The treating psychologist is bound by medical confidentiality under the Health and Medical Services Act. The principal investigator for the study is Professor Catarina Almqvist Malmros, KI, and the contact person is Licensed Psychologist and Medical Doctor Marianne Bonnert (marianne.bonnert@ki.se), mobile phone: 073-748 28 64.

Consent
The next step is to click "I consent" at the bottom of the page, and you will proceed to the questions. If you do not wish to participate, click the yellow box "Cancel registration," and you will be logged out.

By clicking "I consent" below, you acknowledge that you have read through the information in the link above, given your informed consent to participate in the study, and that your personal data will be processed in accordance with the General Data Protection Regulation (GDPR) and the Public Access to Information and Secrecy Act (2009:400).
